# Integral Analysis of the RNA Binding Protein-associated Prognostic Model for Renal Cell Carcinoma

**DOI:** 10.7150/ijms.50704

**Published:** 2021-01-01

**Authors:** Xin Qin, Zhengfang Liu, Keqiang Yan, Zhiqing Fang, Yidong Fan

**Affiliations:** 1 Department of Urology, Qilu Hospital, Cheeloo College of Medicine, Shandong University, Ji'nan, 250012, PR China.; 2 Department of Medicine, Center for Molecular Medicine (CMM) and Bioclinicum, Karolinska Institute and Karolinska University Hospital Solna, Solna 171 64, Sweden.

**Keywords:** Renal cell carcinoma, RNA binding proteins, Overall survival, Prognostic model

## Abstract

RNA binding protein (RBPs) dysregulation has been reported in various malignant tumors and plays a pivotal role in tumor carcinogenesis and progression. However, the underlying mechanisms in renal cell carcinoma (RCC) are still unknown. In the present study, we performed a bioinformatics analysis using data from TCGA database to explore the expression and prognostic value of RBPs. We identified 125 differently expressed RBPs between tumor and normal tissue in RCC patients, including 87 upregulated and 38 downregulated RBPs. Eight RBPs (RPL22L1, RNASE2, RNASE3, EZH2, DDX25, DQX1, EXOSC5, DDX47) were selected as prognosis-related RBPs and used to construct a risk score model. In the risk score model, the high-risk subgroup had a poorer overall survival (OS) than the low-risk subgroup, and we divided the 539 RCC patients into two groups and conducted a time-dependent receiver operating characteristic (ROC) analysis to further test the prognostic ability of the eight hub RBPs. The area under the curve (AUC) of the ROC curve was 0.728 in train-group and 0.688 in test-group, indicating a good prognostic model. More importantly, we established a nomogram based on the selected eight RBPs. The eight selected RBPS have predictive value for RCC patients, with potential applications in clinical decision-making and individualized treatment.

## Introduction

With nearly 73,750 estimated new cases in the US, renal cancer is one of the most common cancers expected to be diagnosed in 2020 1. In turn, the most common type of renal cancer is renal cell carcinoma (RCC), accounting for nearly 80% of renal cancers 2. The definite pathogenesis of renal cancer is still unknown. The certain risk factors include smoking, bodily form and the history of chronic kidney disease 3. Surgical resection, targeted therapy and novel immunotherapy agents have been extensively used in clinics 4. The early diagnosis of renal cancer is difficult and some patients always have distant metastases even at early diagnosis with a poor prognosis 5. Therefore, exploring the valuable prognostic biomarkers is crucial for RCC patients.

RNA-binding proteins (RBPs) play a key role in post‑transcriptional gene regulation (PTGR) 6. RBPs can interact with coding and non-coding RNA by forming ribonucleoprotein (RNP) complexes 7. In a sequence- and structure-dependent manner, RBPs can combine with target RNA to affect mRNA stability, mRNA maturation, splicing, export and translation, thus playing important roles in cell proliferation and differentiation 8. Recent studies have revealed that the deregulation of RBPs are common in many cancers, including myeloid leukemia, breast cancer, hepatocellular carcinoma, colorectal cancer, lung cancer, ovarian cancer, glioblastoma, and bladder cancer 9-18. RBPs regulate multiple signaling pathways involved in tumor proliferation, evasion of cell death, tumor invasion, stemness maintenance, reprograming energy metabolism, and immunity regulation 19. However, the studies of RBPs are rare in RCC, and the roles of RBPs playing in RCC initiation and progression are still unclear.

In our study, RCC RNA-sequencing and clinical information from The Cancer Genome Atlas (TCGA) database were downloaded to perform a systematic functional study. We used R software packages to select differently expressed RBPs between tumor and normal samples and analyze their functions and molecular mechanisms systematically. We identified some prognosis-related RBPs from RCC patients, which can help us understand the molecular mechanisms of renal cell carcinoma and could be the potential biomarkers for diagnosis and prognosis.

## Materials and methods

### Data processing

We downloaded the RNA sequencing dates and clinical information of RCC patients from The Cancer Genome Atlas database (TCGA, http://portal.gdc.cancer.gov/), containing 539 RCC samples and 72 normal renal tissue samples. The R language Limma package (http://www.bioconductor.org/packages/release/bioc/html/limma.html) was used to analyze the data and select the differently expressed RBPs. All raw data was preprocessed with the Limma package and excluded genes with an average count value less than 1. The screening standard was: P<0.05, |log2FC)| >1.0.

### KEGG pathway and GO enrichment analysis

We performed GO and KEGG pathway enrichment analyses to explore the function and mechanisms of differently expressed RBPs. The R packages ClusterProfiler, org.Hs.eg.db, enrichplot and ggplot2 from Bioconductor were used to perform GO enrichment analysis, while the packages ClusterProfiler, colorspace, enrichplot, ggplot2, DOSE and stringi were used to perform the KEGG pathway enrichment analysis. Both p-values and FDR values were statistically significant at less than 0.05.

### PPI network construction and module screening

We submitted the differently expressed RBPs to the STRING database (http://www.string-db.org/) to acquire protein-protein interaction information, and we then used Cytoscape 3.7.0 to construct the PPI network and visualize it. The screening standard was P≤ 0.05.

### Prognostic model construction

The survival and glmnet R packages were used to perform Univariate Cox regression analysis and multiple stepwise Cox regression to construct a risk score model. The risk score formula was as follows:

Risk score = β1*Exp1+ β2*Exp2 …+βi*Expi

β represents the coefficient value. Exp represents the expression of RBPs. The survival ROC R package was used to evaluate the prognostic capability of the above model. The rms R package was used to construct the nomogram. The screening standard was P≤ 0.05.

### Verification of prognostic significance

Gene Expression Profiling Interactive Analysis (GEPIA) (http://gepia.cancer-pku.cn/) database was used to perform survival analysis.

### Gene set enrichment analysis (GSEA)

C2 KEGG gene sets from the Molecular Signatures Database (MSigDB) were used to perform GSEA analysis. The permutation number and recompute time set were both set at 1000. The results should meet our screening criteria (P < 0.05).

### Statistical analysis

The median risk score in each data set was used as a cutoff to compare survival risk between high-risk and low-risk groups, a Kaplan-Meier (KM) curve was drawn. Multivariate Cox regression analysis was used to test whether gene markers were independent prognostic factors. Significance was defined as P < 0.05. All analyses were performed using R 3.4.3.

## Results

### Identification of differently expressed RBPs in RCC patients

**Figure 1:**
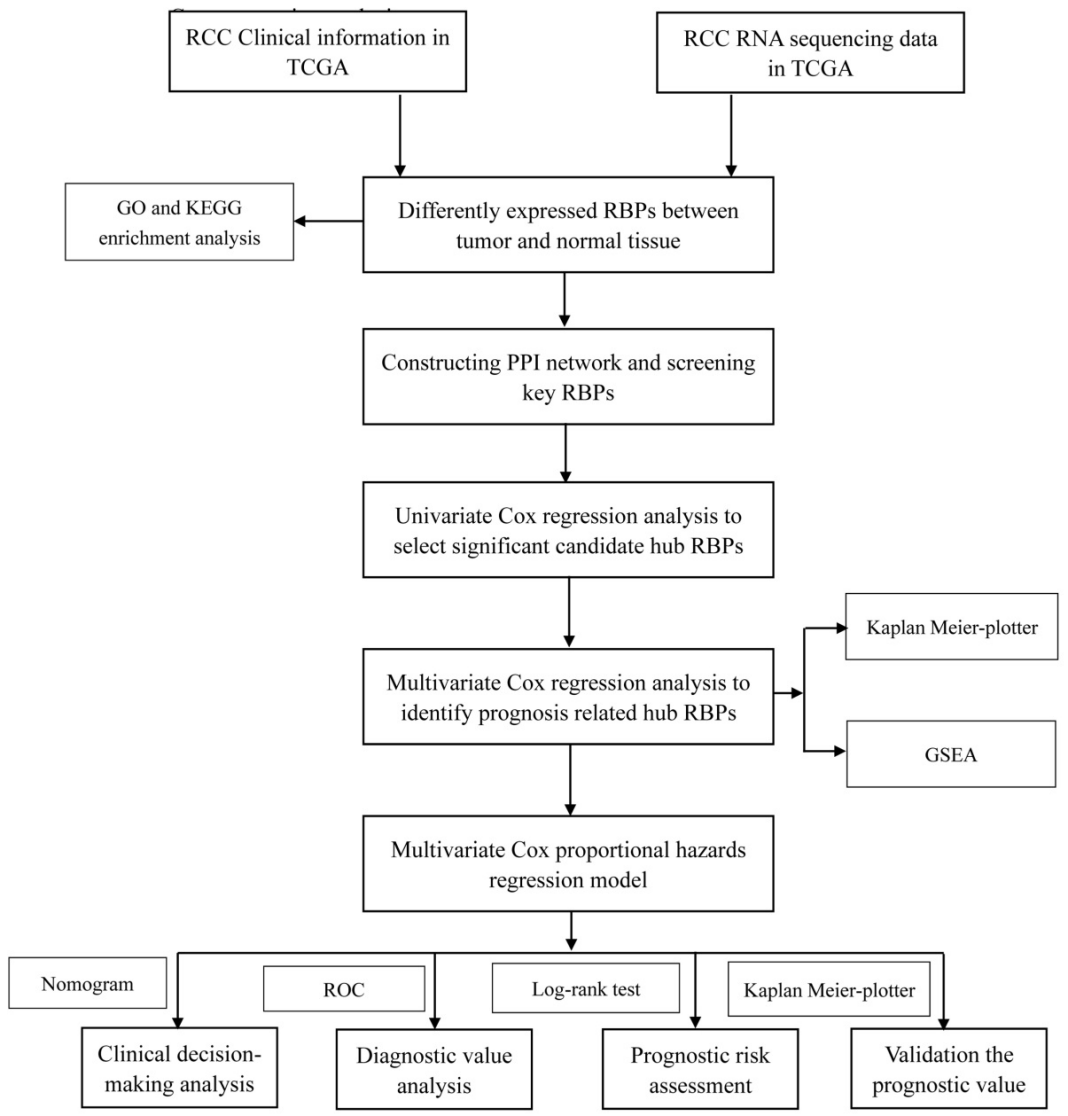
Whole procedures for analyzing RBPs in RCC.

**Figure 2:**
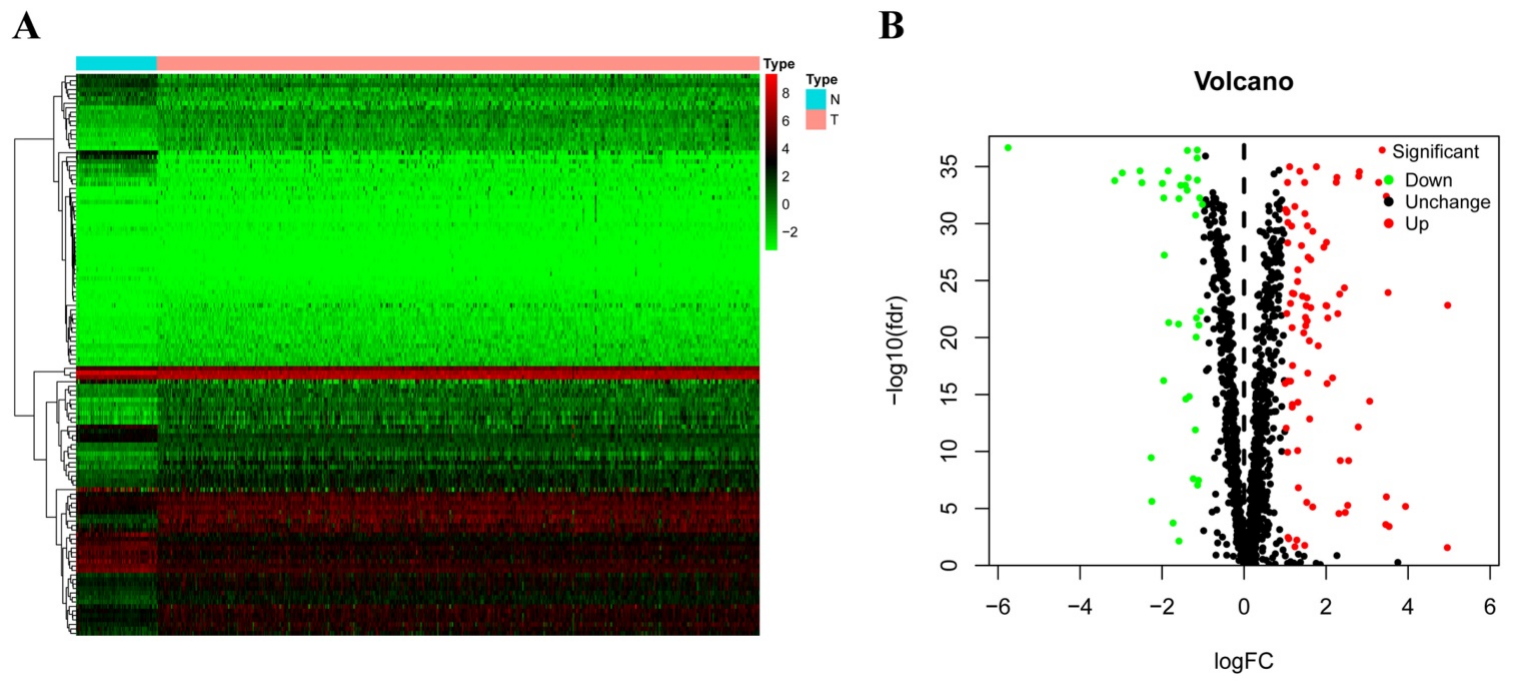
The differently expressed RBPs in RCC. (A) Heat map; (B) Volcano plot.

In this study, we utilized several advanced methods to identify the key prognosis‐related RBPs in RCC patients. The study design was illustrated in Figure 1. The data on RNA sequencing for RCC and corresponding clinical information was sourced from TCGA, with a total of 611 samples: 539 RCC samples and 72 normal renal tissue samples. We used R software packages to analyze the data and select the differently expressed RBPs. We totally analyzed 1542 RBPs 6, and 125 RBPs met our screening criteria (P<0.05, |log2FC)| >1.0), which included 87 upregulated and 38 downregulated RBPs. The expression distribution of these differently expressed RBPs is shown in Figure 2.

### GO and KEGG pathway enrichment analyses of the differently expressed RBPs

**Figure 3:**
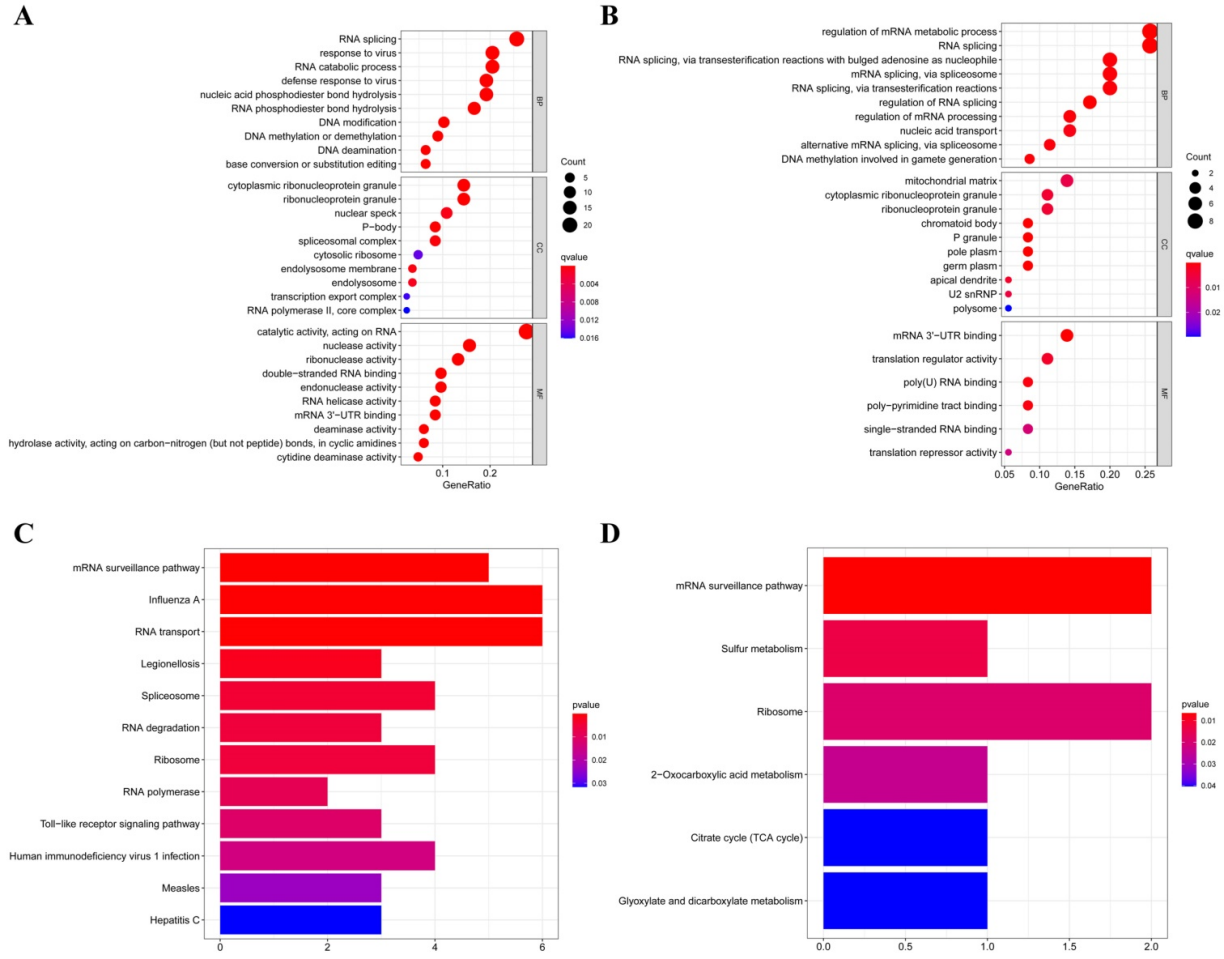
GO and KEGG pathway enrichment analyses of aberrantly expressed RBPs in RCC. (A) GO enrichment analysis of upregulated RBPs; (B) GO enrichment analysis of downregulated RBPs; (C) KEGG pathway enrichment analysis of upregulated RBPs; (D) KEGG pathway enrichment analysis of downregulated RBPs.

According to their different expression levels, we divided these RBPs into two groups (upregulated or downregulated expression) to explore their function and the underlying mechanisms. To assess GO and KEGG pathway enrichment, various R software packages were used to analyze the selected differently expressed RBPs. The results showed that, in biological processes, the upregulated RBPs were notably enriched in RNA splicing, response to viruses, RNA catabolic process and DNA methylation or demethylation (Figure 3A). The downregulated RBPs were significantly enriched in regulation of mRNA metabolic processes, RNA splicing, nucleic acid transport and DNA methylation (Figure 3B). When it comes to the molecular function, the upregulated RBPs were notably enriched in catalytic activity acting on RNA, nuclease activity, ribonuclease activity, double-stranded RNA binding and endonuclease activity (Figure 3A), while the downregulated RBPs were significantly enriched in mRNA 3'-UTR binding, translation regulator activity, poly(U) RNA binding and poly-pyrimidine tract binding (Figure 3B). As for the cellular component, we found that the upregulated RBPs were mainly enriched in ribonucleoprotein granules, nuclear specks, P-bodies and spliceosomal complexes (Figure 3A), while the downregulated RBPs were mainly enriched in mitochondrial matrices, ribonucleoprotein granules, chromatoid bodies and P granules (Figure 3B). Moreover, we found that the upregulated RBPs were mainly enriched in mRNA surveillance pathways, Influenza A, RNA transport, spliceosomes and RNA degradation (Figure 3C), and the downregulated RBPs were mainly enriched in mRNA surveillance pathways, sulfur metabolism, ribosome and 2-oxocarboxylic acid metabolism (Figure 3D).

### PPI Network Construction and Key Module Screening

**Figure 4:**
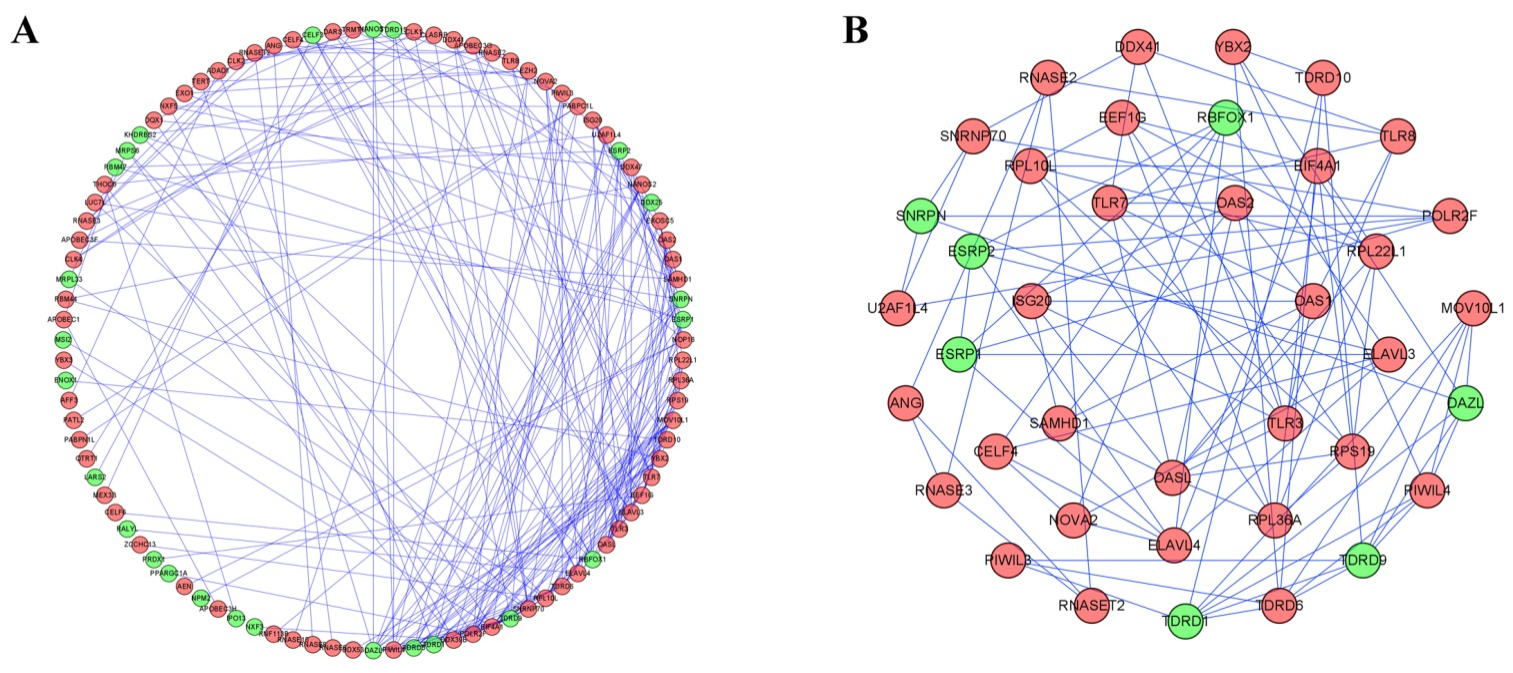
Protein-protein interaction network and modules analysis. (A) PPI network of differently expressed RBPs; (B) Critical module from PPI network. Green circles: downregulation; red circles: upregulation.

To better understand the correlation and function of these differently expressed RBPs in RCC patients, we built a PPI network by using Cytoscape software and the STRING database, which included 100 nodes and 225 edges (Figure 4A). By using the MODE tool in Cytoscape to process the PPI network, we obtained the most important module which contained 39 nodes and 89 edges (Figure 4B). The RBPs in this module were greatly enriched in ribosome assembly, cytoplasmic translation, regulation of mRNA processing, regulation of RNA splicing, piRNA metabolic process, DNA methylation, meiotic cell cycle and RNA transcription.

### Prognosis-Related RBP Screening

**Figure 5:**
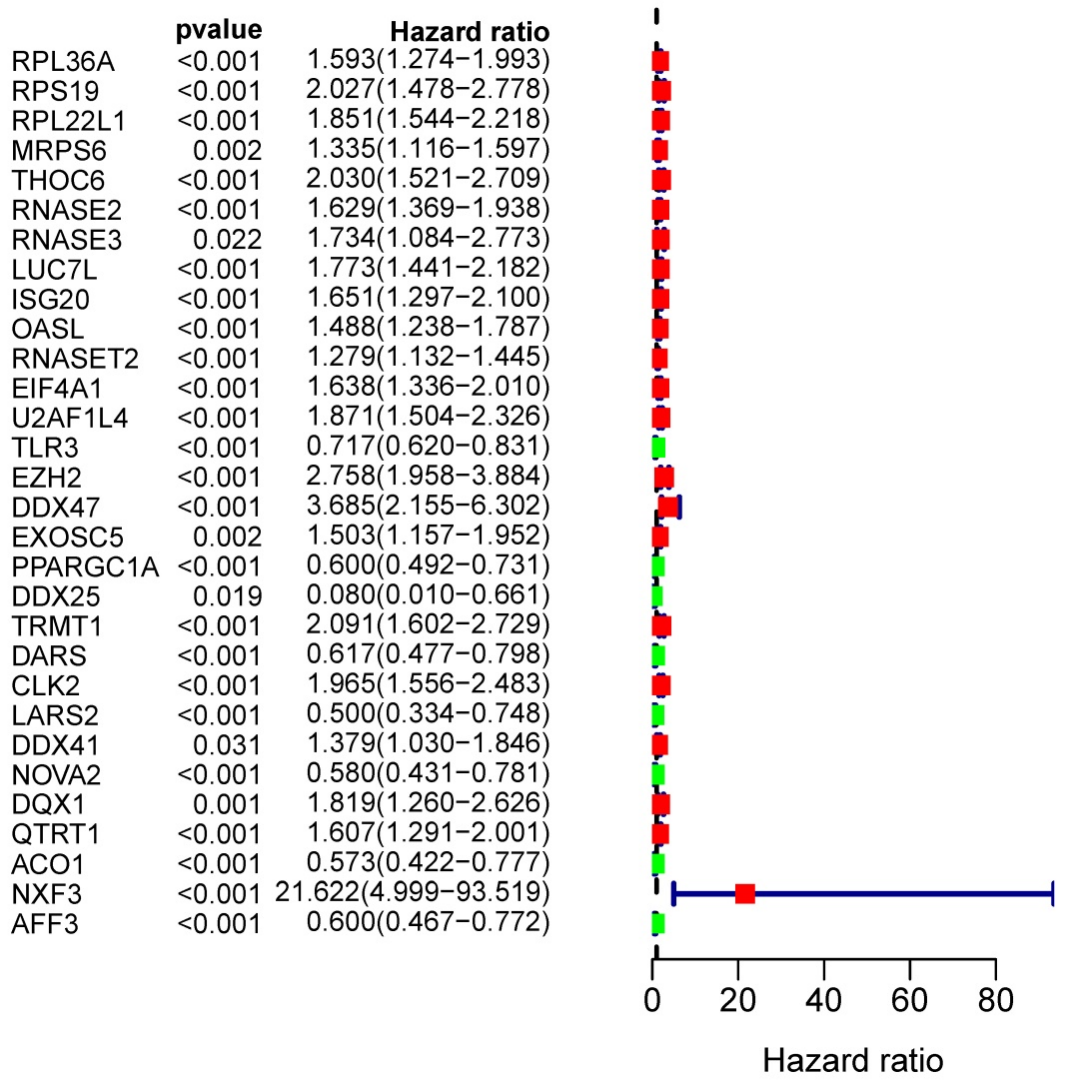
Univariate Cox regression analysis to identify hub RBPs.

**Figure 6:**
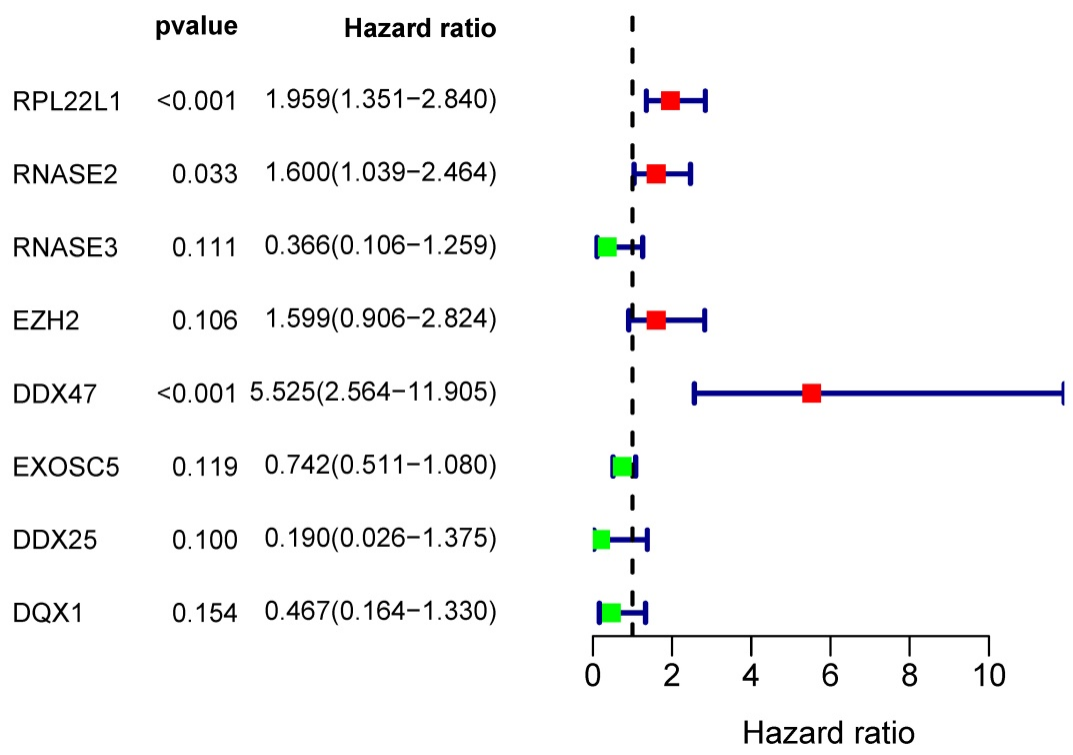
Multivariate Cox regression analysis for identification of prognosis-related hub RBPs.

From the PPI network, we uncovered 100 differently expressed RBPs in total. By univariate Cox regression analysis, we obtained 30 prognostic-associated candidate RBPs (Figure 5). Then we performed a multiple stepwise Cox regression and selected eight hub RBPs to be independent predictors in RCC patients (Figure 6).

### Prognosis-Related Genetic Risk Score Model Construction and Validation

First, we randomly divided the RCC patients into a train-group and test-group. In the train-group, we used the eight hub RBPs selected by the multiple stepwise Cox regression to build the predictive model. Then we used a formula to calculate the risk score of each RCC patient. The formula is as follows:

Risk score = (0.6724*ExpRPL22L1) + (0.4699*ExpRNASE2) + (-1.0050*ExpRNASE3) + (0.4695*ExpEZH2) + (1.7092*ExpDDX47) + (-0.2978*ExpEXOSC5) + (-1.6594*ExpDDX25) + (-0.7605*ExpDQX1)

**Figure 7:**
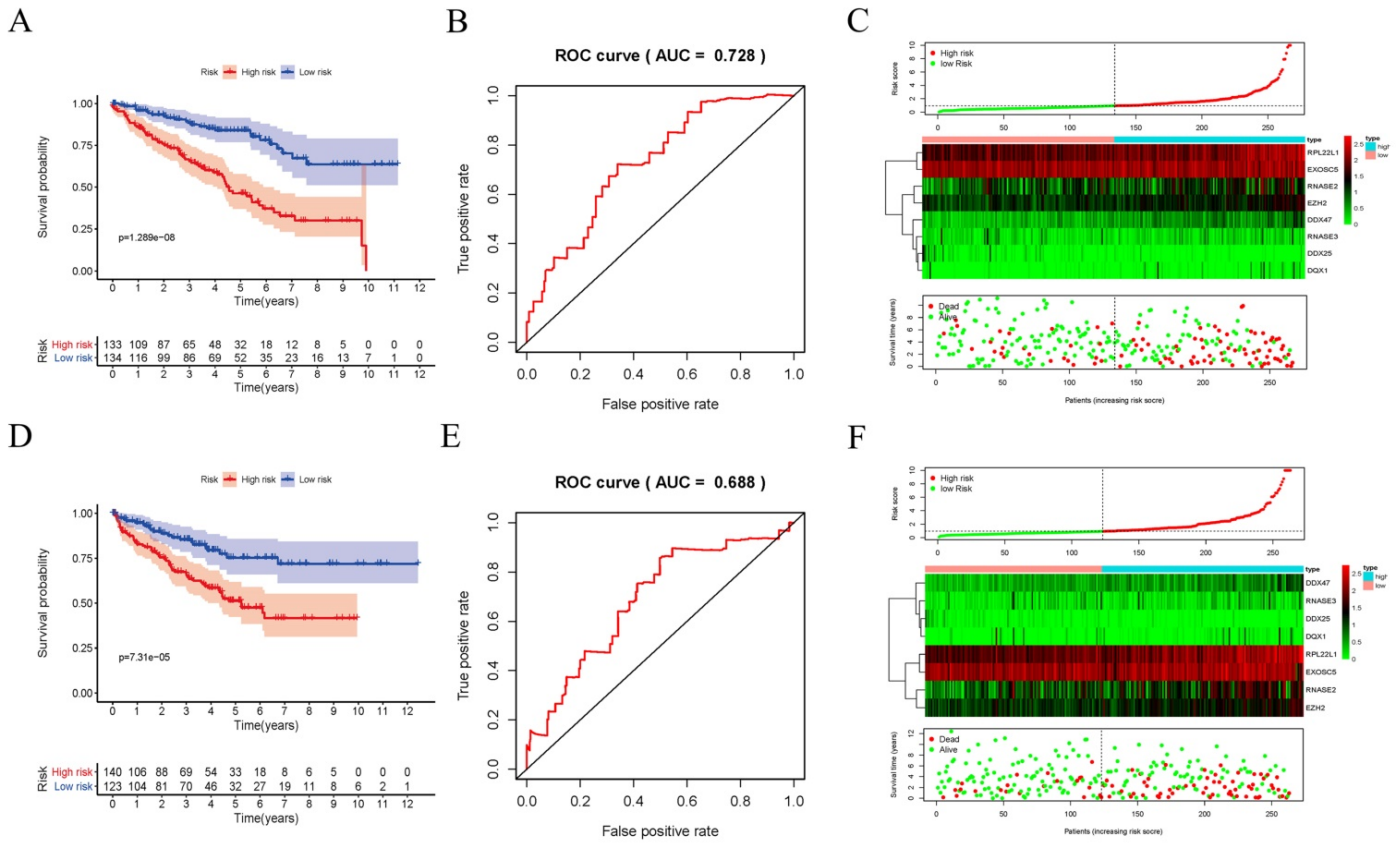
Risk score analysis of risk model in train-group and test-group. (A) Survival curve for low- and high-risk subgroups in train-group; (B) ROC curves for forecasting OS in train-group; (C) Risk score, expression heat map and survival status in train-group; (D) Survival curve for low- and high-risk subgroups in test-group; (E) ROC curves for forecasting OS in test-group; (F) Risk score, expression heat map and survival status in test-group.

**Figure 8:**
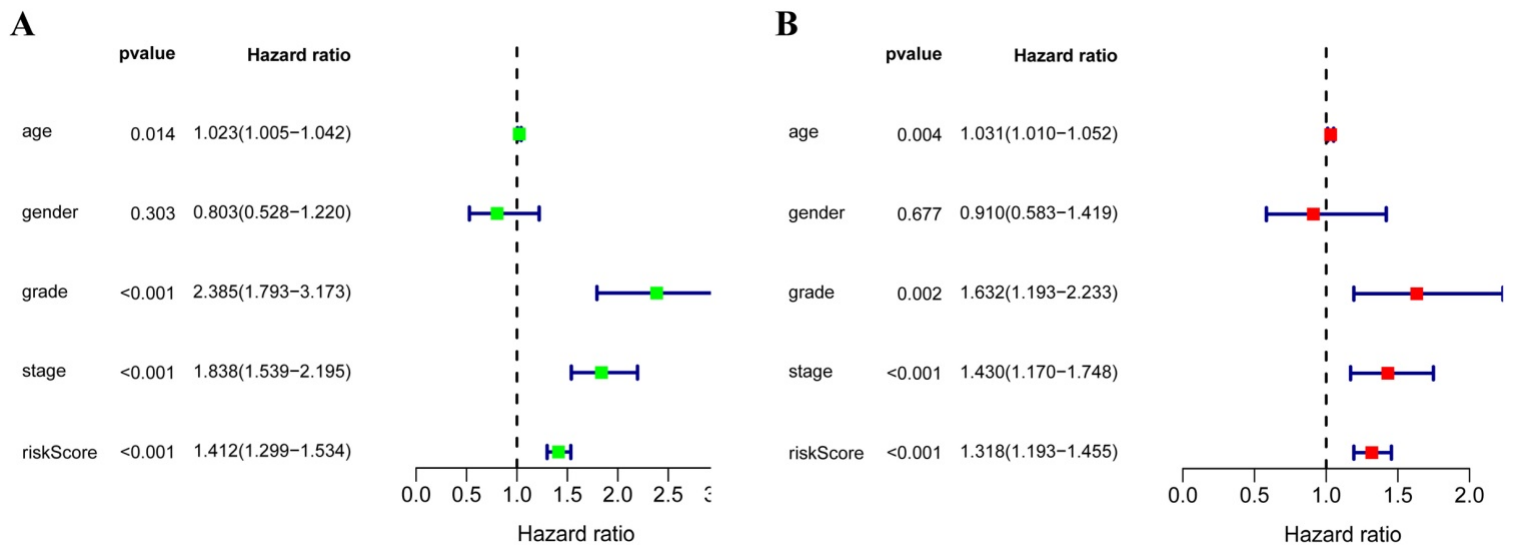
(A) Univariate Cox regression analysis for different clinical parameters; (B) Multivariate Cox regression analysis for different clinical parameters.

In order to evaluate the predictive ability of this model, 267 RCC patients were divided into two subgroups according to the median risk score. 133 high-risk patients and 134 low-risk patients were analyzed to perform a survival analysis. The results showed that the high-risk subgroup had a poorer OS than the low-risk subgroup (Figure 7A). Second, we conducted a time-dependent ROC analysis to further test the prognostic ability of the eight hub RBPs. The AUC of the ROC curve was 0.728, indicating a moderate clinical prognostic significance (Figure 7B). The risk score, survival status of patients and expression heat map of the two subgroups are shown in Figure 7C. To prove the accuracy of the risk score model, we used the data of the test-group to perform the same analysis. The result was similar (Figure 7D-F), suggesting that the risk score model is reliable. To explore the relationship of clinical features with our predictive model, we then performed a regression analysis. Univariate regression analysis indicated age, tumor grade, tumor stage and risk score were relevant to OS of RCC patients (Figure 8A). Multiple regression analysis further showed that age, tumor grade, tumor stage and risk score were also independent prognostic factors related to OS (Figure 8B).

### Construction of a nomogram based on Risk Score Model

**Figure 9:**
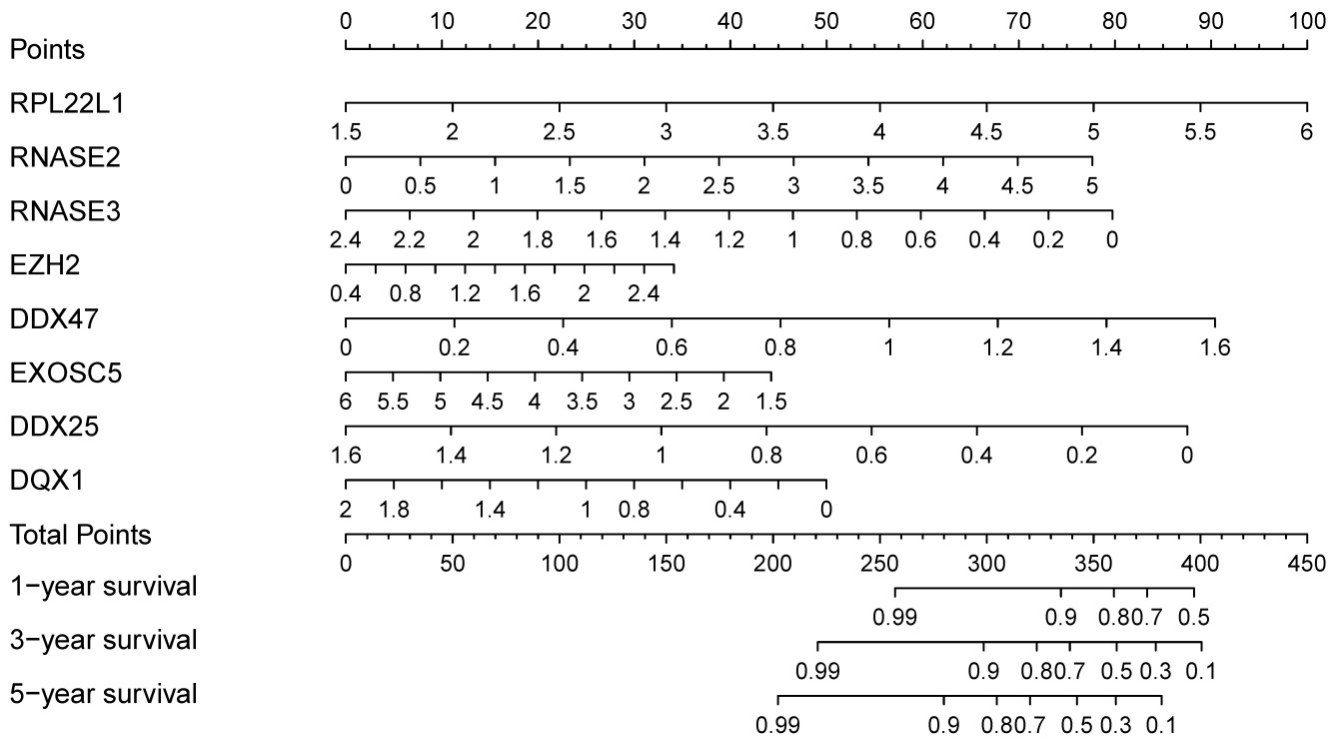
Nomogram for predicting OS of RCC patients.

**Figure 10:**
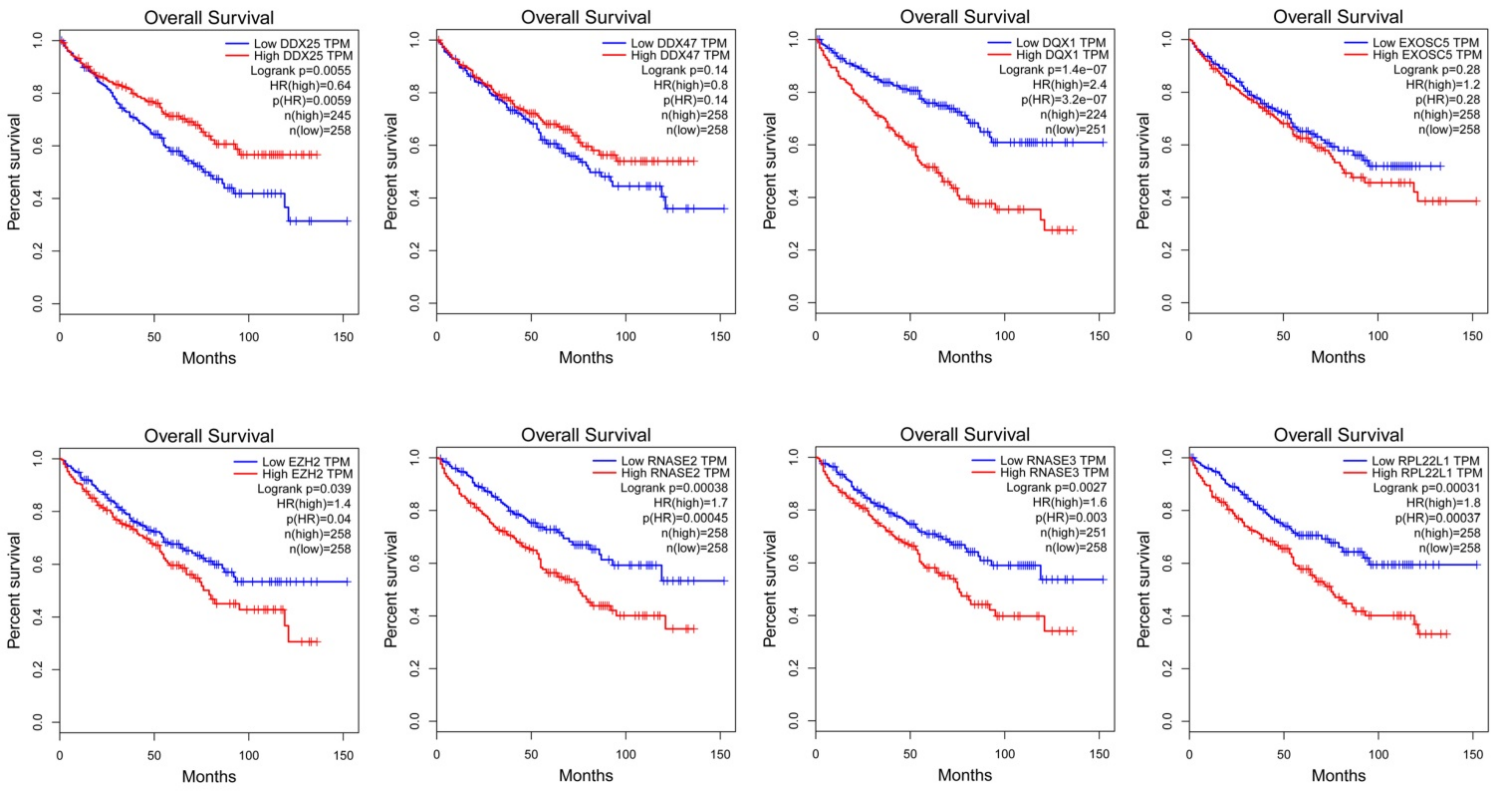
Validation of the prognostic value of hub RBPs in RCC patients by Kaplan Meier-plotter.

**Figure 11:**
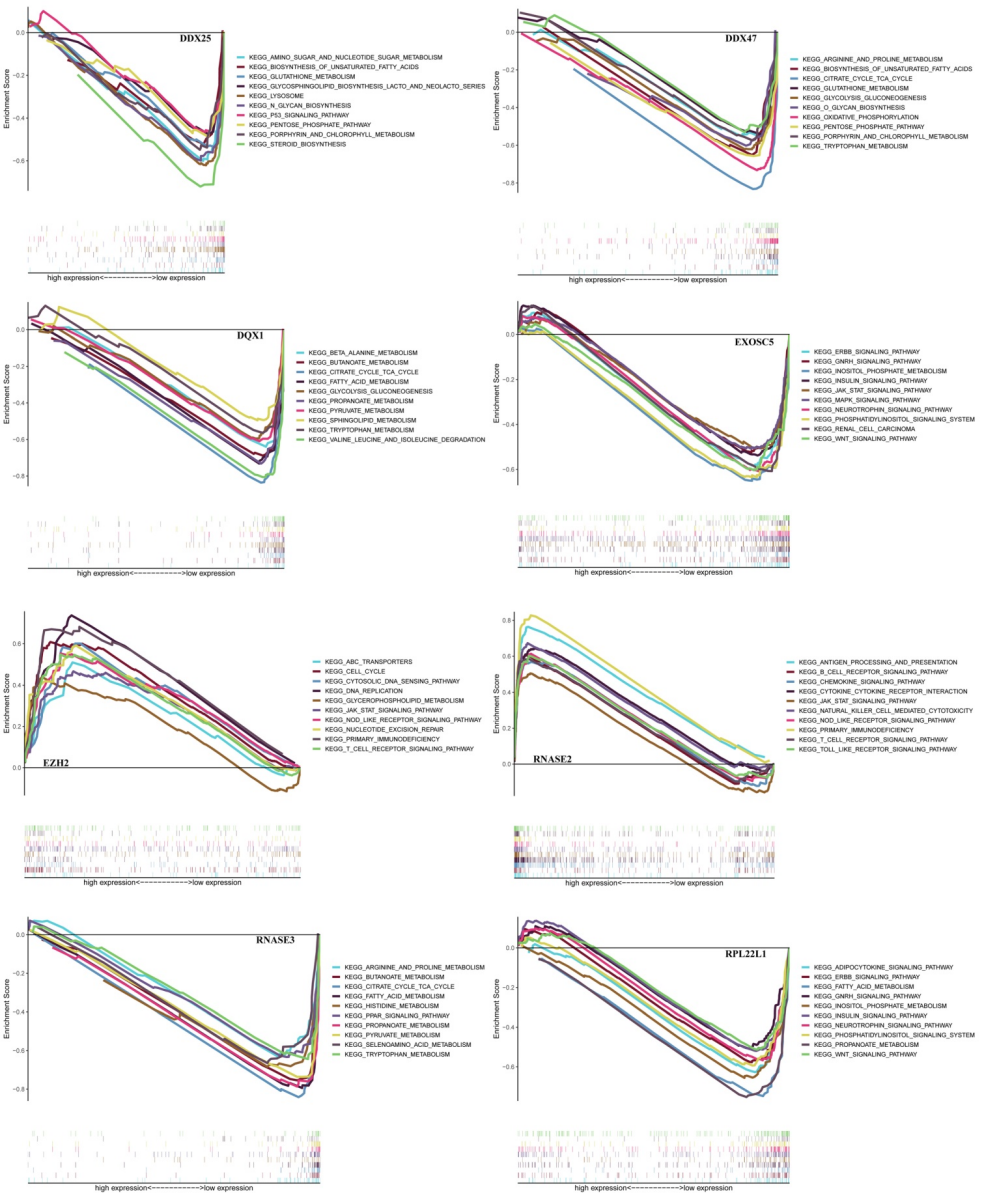
GSEA analysis of eight hub RBPs with C2 KEGG gene sets.

In order to find a quantitative method for RCC prognosis, we combined the eight-RBPs biomarkers to build a nomogram (Figure 9). By calculating the point of each variable and taking all points into account, we could predict the estimated survival rates for RCC patients at one, three, and five years, and the nomogram can help us to make clinical decisions for RCC patients. To further explore the prognostic value of the eight hub RBPs, we performed a survival analysis based on the data from the GEPIA database to explore the relationship between the hub RBPs and OS. The results show that six RBPs (RPL22L1, RNASE2, RNASE3, EZH2, DDX25, DQX1) were related with the OS (Figure 10). To further explore the functions of these selected RBPs, C2 KEGG gene sets from the Molecular Signatures Database (MSigDB) were used to perform a single gene GSEA analysis. The results were shown in Figure 11, DDX47, DDX25, DQX1 and RNASE3 are mainly enriched in synthesis and metabolism of glucose, protein and fat. EXOSC5 is involved in various signaling pathways, such as JAK-STAT signaling pathway, MAPK signaling pathway and WNT signaling pathway. RNASE2 may relate with immunomodulation, such as antigen processing and presentation, B cell receptor signaling pathway, natural killer cell mediated cytotoxicity and T cell receptor signaling pathway. EZH2 was significantly enriched in the regulation of cell cycle, DNA damage repair, JAK-STAT signaling pathway and WNT signaling pathway. While RPL22L1 regulated propanoate metabolism, fatty acid metabolism, adipocytokine signaling pathway and insulin signaling pathway.

## Discussion

The dysregulated expression of RBPs has been reported to play a pivotal role in tumor carcinogenesis and progression in many malignant tumors 19. However, the underlying mechanisms are still unclear 19. In our study, we downloaded the information of RCC patients from TCGA. Though bioinformatics analysis, we selected 125 differently expressed RBPs in RCC between tumor and normal tissue, and we performed a detailed and systematic analysis to explore their functions and pathway enrichment. Moreover, we built a co-expression network and PPI network of these differently expressed RBPs. Then, by univariate Cox regression analysis and multiple stepwise Cox regression, we identified eight hub RBPs and constructed a risk model to predict prognosis. To verify and further explore the prognostic value of our model and expression of hub RBPs, we performed ROC analysis, risk score, survival status of patients, expression heat map and survival analysis. Our findings helped us to establish potential biomarkers for RCC patients' diagnosis and prognosis.

GO enrichment analysis showed that the differently expressed RBPs were greatly enriched in regulation of mRNA metabolic processes, RNA splicing, nucleic acid transport, RNA catabolic processes and DNA methylation. Previous studies have showed that RBPs can regulate the occurrence and development of many diseases via post-transcriptional gene regulation 20-22. The KEGG pathways analysis showed that the dysregulated RBPs were mainly enriched in mRNA surveillance pathways, RNA transport, spliceosome and RNA degradation.

Subsequently, we constructed a co-expression network and PPI network and selected 100 key RBPs, which play a key role in the disease progression. Among these key RBPs, many of them have been studied and found to play the important roles in the development and progression of other tumors via regulating the expression of oncoproteins and tumor-suppressor proteins 19. Moreover, we used the MODE tool in Cytoscape to get important subnetworks. The results showed that these modules were greatly enriched in the regulation of mRNA processing, RNA splicing, piRNA metabolic processes, DNA methylation and RNA transcription.

Additionally, via univariate Cox regression analysis, survival analyses, and multiple Cox regression analysis, we identified eight hub RBPs (RPL22L1, RNASE2, RNASE3, EZH2, DDX25, DQX1, EXOSC5, DDX47) and built a risk model to predict prognosis of RCC. The ROC curve analysis showed that our model is significant and sensitive, and it will be of value to guide treatment and prognosis for RCC patients. These eight RBPs function either as oncogenes or tumor-suppressor genes in cancers. Through regulating the expression of MGMT and MLH1, RPL22L1 can affect tumor progression and chemotherapeutic drug resistance 23 RNASE2 and RNASE3 are mainly involved in human immune function, and RNASE2 plays an important role in sensing of pathogens by toll-like receptor 8 (TL8) 24. Hence, researchers have identified that combination analysis of RNASE2 with other 6 immune-related genes has prognostic value in ccRCC 25. EZH2, the core enzymatic subunits of PRC2, plays a pivotal role in tumor carcinogenesis and progression via modulating many pro-oncogenic and pro-survival signaling pathways 26. Moreover, the expression of EZH2 significantly increases in RCC tissue than that in normal tissue associated with a poor outcome, and EZH2 promotes sunitinib resistance in RCC through kinome reprogramming 27. DDX47 is involved in maintenance of genome stability via interacting with FANCD2 to lower R-loop levels 28, and study has revealed that overexpression of DDX47 induces tumor cells apoptosis 29. DQX1, acting as the methylation-driven genes, is a novel prognostic marker in lung squamous cell carcinoma 30. EXOSC5 could promotes growth of colorectal cancer through regulating ERK and AKT pathways 31. Collectively, the studies of these eight RBPs in RCC are rare, and the underlying molecular mechanism is still obscure and deserves further exploration.

Overall, our risk model only needs to detect eight hub genes, and it is economical and acceptable for RCC patients. Our analysis also showed that the eight hub RBPs have their own important biological functions and are associated with patients' prognoses, indicating that they have potential to be used for clinical assistance treatment. However, our study did of course have several limitations. First, our data only comes from TCGA database, and is not validated in clinical patient cohorts. Second, our data is only based on RNA sequencing without verification via other omics data platforms. Lastly, the loss of some clinical information reduces the accuracy of multivariate stepwise Cox regression analysis.

In summary, we have systemically analyzed the expression and function of RBPs in RCC patients and acquired the diagnostic model and independent prognostic factor for RCC patients. To our knowledge, this is the first report on the RBP prognosis-associated model for RCC patients, and our study shows that RBPs could be prognostic markers as well as new targets for RCC patients.
